# Long-term biochemical stability of fresh-frozen plasma from Asian elephants (*Elephas maximus*) stored at −20°C: Implications for emergency transfusion protocols for elephant endotheliotropic herpesvirus hemorrhagic disease

**DOI:** 10.14202/vetworld.2026.125-134

**Published:** 2026-01-14

**Authors:** Chatchote Thitaram, Pakkanut Bansiddhi, Araya Pakamma, Kontawan Arintasai, Siripat Khammesri, Chonticha Sirikul, Worapong Kosaruk, Janine L. Brown, Preeyanat Vongchan

**Affiliations:** 1 Center of Elephant and Wildlife Health, Faculty of Veterinary Medicine, Chiang Mai University, Thailand; 2 Elephant, Wildlife, and Companion Animals Research Group, Chiang Mai University, Chiang Mai, Thailand; 3 Department of Medical Technology, Faculty of Associated Medical Sciences, Chiang Mai, Thailand; 4 Center for Species Survival, Smithsonian National Zoo and Conservation Biology Institute, Front Royal, Virginia, United States

**Keywords:** Asian elephant, coagulation factors, EEHV hemorrhagic disease, factor VIII activity, fibrinogen stability, fresh-frozen plasma, plasma banking protocols, wildlife transfusion medicine

## Abstract

**Background and Aim:**

Elephant endotheliotropic herpesvirus hemorrhagic disease (EEHV-HD) is a leading cause of fatal hemorrhagic illness in juvenile Asian elephants (*Elephas maximus*), often requiring urgent plasma transfusion. However, the biochemical stability of fresh-frozen plasma (FFP) during long-term storage has not been systematically evaluated in this species. This study assessed the stability of key plasma proteins, fibrinogen, clotting factor VIII, immunoglobulin G (IgG), and albumin, in FFP stored at −20°C for 4, 8, and 12 months, and compared them with fresh plasma to determine suitability for emergency clinical use.

**Materials and Methods:**

Plasma samples were collected from 20 healthy elephants and processed into fresh and frozen aliquots. Fibrinogen concentrations were quantified using the Clauss assay, factor VIII activity via a one-stage clotting assay, and IgG and albumin concentrations using colorimetric methods. A repeated-measures generalized linear model evaluated the effects of storage duration on protein stability, with post hoc Tukey adjustments.

**Results:**

Fibrinogen concentrations remained stable during storage, with no significant differences at 8 or 12 months compared with fresh plasma. Factor VIII activity declined progressively, with a significant 16% reduction after 12 months (p < 0.001), though values remained within clinically acceptable ranges. Conversely, IgG and albumin concentrations increased significantly during frozen storage, with 37% and 21% higher values, respectively, at 12 months, likely reflecting cryoconcentration. Neither sex nor other covariates significantly influenced protein stability.

**Conclusion:**

This study provides the first evidence that elephant FFP stored at −20°C retains acceptable biochemical stability for up to 12 months. Although factor VIII activity decreases over time, fibrinogen remains stable, and immunoproteins increase, supporting the clinical utility of stored plasma in EEHV-HD emergencies. These findings provide foundational guidance for establishing elephant plasma banking protocols, improving readiness for rapid intervention, and advancing One Health–aligned conservation strategies for endangered megafauna.

## INTRODUCTION

Elephant endotheliotropic herpesvirus (EEHV) remains a major cause of mortality in juvenile *Elephas maximus* (Asian elephants), particularly those between 1 and 8 years of age. The virus causes an acute, often rapidly fatal hemorrhagic disease and is recognized as one of the leading causes of death in young elephants [1, 2]. Since Richman *et al*. [3] first identified EEHV in 1999, more than 100 fatal cases have been documented worldwide, with EEHV1 and EEHV4 the most commonly implicated types [4]. Early clinical signs are frequently nonspecific, including lethargy, anorexia, and fever, before progressing to more characteristic features such as facial and cervical edema and tongue cyanosis. Without intervention, these signs can progress to fulminant hemorrhage, with death occurring within 1–7 days of onset [5, 6]. Some calves deteriorate even more rapidly; Sripiboon *et al*. [7] reported fatalities within 12 h, with a median survival time of only 36 h once clinical signs appear [8]. Mortality rates are extremely high (80%–90%) [9], and even with antiviral therapy, survival typically remains around 10% [4]. However, recent advances have reduced mortality to 66% [10, 11], and survival may improve to as low as 19% when aggressive treatment is initiated within the first 24 h [12]. The disease is characterized by widespread hemorrhage and subsequent hypovolemic shock, making rapid diagnosis and intervention critical. Current treatment protocols employ antiviral medications, such as acyclovir, famciclovir, and ganciclovir, together with fluid therapy and plasma transfusion [13].

Plasma transfusion plays a pivotal therapeutic role by supporting rehydration, restoring plasma protein levels, enhancing immunological defense, mitigating hypovolemic shock, and stabilizing blood pressure. Clinical cases illustrate its value: Angkawanish *et al*. [14] successfully used whole blood and plasma transfusion in an elephant with severe postpartum hemorrhage, and another debilitated elephant with prolonged esophageal obstruction responded positively to transfusion therapy (Langkaphin, personal communication). Fresh plasma is particularly recommended for EEHV-HD because it provides antibodies, coagulation factors, and platelets needed to counteract hemorrhagic pathology. However, preparing fresh plasma is time-intensive, requiring donor selection, health screening, infectious disease testing, and cross-matching, often compounded by geographic and logistical challenges [15]. These constraints underscore the need for an immediately available alternative. Fresh-frozen plasma (FFP) provides a practical solution, enabling timely administration during emergencies and highlighting the urgent need for plasma therapy preparedness in endangered elephant populations.

FFP is produced by collecting whole blood into anticoagulant-containing containers, separating the plasma, and freezing it for long-term storage. It contains key proteins, including albumin, globulins, and coagulation factors I–XIII, and retains approximately 70% of its original factor VIII activity. In human medicine, FFP is widely used to treat coagulation disorders such as disseminated intravascular coagulation (DIC) [16], a condition also reported in EEHV-HD [17–19]. FFP also supports the management of severe hemorrhage in both humans and animals [20], enhances immunoglobulin (Ig)G levels in foals with failure of passive transfer [21], and supplies coagulation factors, albumin, α-macroglobulin, and Igs in dogs [22]. Recently, species-specific plasma protocols have been developed for diverse exotic animals, including macaques and marine mammals [23–25].

Storage duration and temperature markedly influence the stability of clotting factors in FFP. Wardrop and Brooks [26] demonstrated considerable variability in factors II, VII, VIII, IX, X, and von Willebrand factor (vWf) in canine FFP stored under different conditions. Alesci *et al*. [27] reported that lower storage temperatures (−70°C and −196°C) better preserved coagulation activity than −20°C. Similarly, Woodhams *et al*. [28] found that samples stored at −24°C remained stable for 3 months, whereas those at −74°C remained stable for at least 18 months.

Fibrinogen, an essential glycoprotein, is key to hemostasis by promoting clot formation and preventing blood loss. Factor VIII plays a central role in the coagulation cascade, whereas IgG is crucial for humoral immunity. Quantifying these proteins is essential for assessing FFP quality. Species-specific differences in plasma composition mean that stability profiles observed in domestic animals cannot be directly extrapolated to elephants. Therefore, evaluating fibrinogen, factor VIII, and IgG stability during frozen storage is essential for establishing evidence-based plasma banking protocols. Progress in wildlife transfusion medicine underscores the importance of species-specific approaches [23, 29–31], enabling improved plasma preservation, handling, and field deployment in conservation medicine [32].

Although plasma transfusion has become an essential therapeutic tool for managing hemorrhagic and hypovolemic conditions in wildlife and domestic species, significant knowledge gaps remain regarding the biochemical stability and clinical readiness of FFP specifically for Asian elephants. Existing literature provides foundational insights into plasma use in other species, yet none address the unique physiological attributes of elephants or the demands of rapid-response treatment required during EEHV-HD outbreaks.

Current evidence in equines shows that plasma transfusion can improve neutrophil function and immune competence in compromised foals, underscoring the importance of functional Igs in transfused plasma [21]. Similarly, canine studies indicate that FFP provides critical coagulation proteins, albumin, α-macroglobulin, and Igs, making it a vital supportive therapy during severe disease or trauma [22]. These findings suggest that stored plasma must retain sufficient immunological and hemostatic components to be clinically effective. However, such conclusions are derived from domestic animals with well-established transfusion systems, not megafauna.

Further, pathological and molecular studies in elephants highlight the rapid hemodynamic deterioration and DIC-like changes associated with EEHV-HD, including severe coagulation dysfunction and depletion of blood proteins [19]. This underscores the clinical need for plasma products that maintain stable coagulation factor activity after storage. Yet despite plasma’s therapeutic importance, no research has evaluated whether elephant FFP stored at standard wildlife facility temperatures (−20°C) retains adequate biochemical integrity over time.

In addition, recent advances in exotic species transfusion medicine, including optimized platelet-rich plasma protocols and species-specific adaptations for marine mammals, demonstrate that specialized transfusion strategies can significantly improve clinical outcomes [23]. However, these innovations remain species-specific and cannot be extrapolated to elephants, whose plasma composition, clotting factor profiles, and storage resilience remain uncharacterized.

Collectively, these studies reveal a critical gap: there is no empirical evidence evaluating the long-term stability of fibrinogen, factor VIII, IgG, or albumin in frozen plasma from Asian elephants, despite their essential roles in EEHV-HD therapy. Without baseline data on how storage duration affects plasma protein viability, wildlife hospitals cannot establish evidence-based plasma banking protocols or ensure that stored FFP remains therapeutically effective during emergencies.

The primary aim of this study was to systematically evaluate the biochemical stability and functional suitability of FFP derived from Asian elephants when stored at −20°C for extended periods commonly used in wildlife facilities. Given the critical role of plasma transfusion in managing EEHV-HD, a condition characterized by rapid-onset coagulopathy, hypoproteinemia, and high mortality, there is an urgent need to establish evidence-based plasma banking protocols that ensure timely therapeutic readiness. To address this need, the study specifically assessed key plasma constituents essential for hemostasis and immune support, including fibrinogen, clotting factor VIII, IgG, and albumin, comparing their concentrations in fresh plasma with those after 4, 8, and 12 months of storage. By quantifying the extent to which each protein is preserved or degraded during storage, this research sought to determine whether elephant FFP retains sufficient functional integrity to be effectively used in emergency clinical interventions for EEHV-HD and other hemorrhagic conditions. Additionally, the study aimed to generate foundational data that support the development of species-specific transfusion guidelines, enhance clinical decision-making in wildlife hospitals, and contribute to establishing standardized plasma banking systems for endangered megafauna within One Health and conservation medicine frameworks.

## MATERIALS AND METHODS

### Ethical approval

All procedures involving animals in this study were conducted in full compliance with national and institutional guidelines governing the ethical use of animals in research. The study protocol, including animal selection, handling, blood collection techniques, and sample processing, was reviewed and approved by the Institutional Animal Care and Use Committee of the Faculty of Veterinary Medicine, Chiang Mai University, Thailand (Approval No. FVM-ACUC; S8/2561). This approval confirms that the study adhered to the ethical principles outlined in the Animal Research: Reporting of *In Vivo* Experiments 2.0 guidelines and followed standards designed to minimize discomfort, stress, and risk to participating animals.

All elephants enrolled in the study were privately owned and housed in elephant camps that routinely collaborate with Chiang Mai University for health monitoring and welfare management. Before sampling, each elephant underwent a physical examination by a licensed veterinarian to confirm eligibility and ensure no underlying health conditions or infectious diseases were present. Blood samples were collected by trained veterinarians or experienced elephant handlers using minimally invasive techniques. Positive reinforcement (food rewards) was used to reduce stress during handling, and no sedation or coercive methods were used. The entire sampling process was completed within a short time frame to minimize disturbance.

The study did not involve experimental treatments, drug administration, or procedures likely to cause pain or long-term physiological effects. Only the volume of blood permitted under institutional welfare guidelines for large mammals was collected, and all animals returned immediately to their normal husbandry routines after sampling. No elephant experienced adverse effects during the study.

This ethical approval ensures that the work was conducted with the utmost regard for animal welfare, scientific integrity, and regulatory compliance, recognizing the cultural, conservation, and ecological importance of Asian elephants.

### Animals

A total of 20 healthy Asian elephants, 10 males and 10 females, were selected from two elephant camps in Chiang Mai, Thailand. Elephants showing signs of illness or infection were excluded from the study. The animals ranged in age from 10 to 60 years. All elephants were fed a diet primarily of corn stalks and Napier grass (*Pennisetum purpureum*) and had unrestricted access to fresh water. They regularly participated in tourist-related activities, including saddle riding, bathing, and feeding. Physical examinations conducted by camp veterinarians confirmed their good health at the time of sampling.

### Blood collection and handling

Blood samples were collected once from an ear vein by trained elephant camp staff or veterinarians from Chiang Mai University in April 2018. Using a 1-inch, 18-gauge IV catheter and a 50-mL syringe, 30 mL of blood was collected between 10:00 a.m. and 12:00 p.m. Each collection was completed within 5 min, with elephants calmly restrained using positive reinforcement (food rewards). Fourteen milliliters of blood were transferred into 50-mL conical tubes preloaded with citrate phosphate dextrose (CPD) anticoagulant (C7165, Sigma-Aldrich, MO, USA) at a 1:7 ratio (2 mL CPD:14 mL blood). The remaining 16 mL were placed into red-top tubes (BD Vacutainer® Serum, USA) without anticoagulant and left undisturbed for 2 h to allow serum separation. All tubes were clearly labeled, kept on ice at approximately 4°C in a Styrofoam container, and transported immediately for processing within 4 h of collection.

### Plasma and serum preparation

Upon arrival at the laboratory, anticoagulated blood was centrifuged at 700 × *g* for 10 min at 4°C (Eppendorf 5810R, Germany) to obtain plasma, yielding 55%–60% of total blood volume. Plasma was aliquoted into four microcentrifuge tubes (2 mL each). Serum tubes were centrifuged at 252 × *g* for 10 min, yielding 40%–45% serum, which was aliquoted into eight microcentrifuge tubes (1 mL each).One plasma and two serum aliquots were analyzed immediately, while the remaining samples were stored at −20°C for analysis at 4, 8, and 12 months with temperature monitoring. Prior to testing, frozen plasma and serum were thawed in a 37°C water bath and analyzed within 2 h. Samples showing hemolysis or lipemia were excluded.

Plasma was used for fibrinogen and factor VIII assays because it contains intact clotting factors, which are preserved by anticoagulants. Serum, which lacks clotting factors due to coagulation during processing, was used for IgG and albumin assays to minimize assay interference.

### Biochemical assays

#### Fibrinogen and factor VIII assays

Plasma samples were submitted to the hematology laboratory, Faculty of Medicine, Chiang Mai University. Fibrinogen concentrations were measured using the Clauss assay (Sysmex CS-2500®, Kobe, Japan) as described by Mackie *et al*. [33]. Internal quality control was performed using low- and high-control commercial plasmas. Intra- and inter-assay coefficients of variation were 2.65% and 4.5%, respectively. For fibrinogen determination, 100 NIH units/mL bovine thrombin (Sigma-Aldrich) were added to 1:10-diluted plasma, and clotting time and prothrombin time (PT) were compared with a calibration curve derived from standard plasma dilutions. Factor VIII activity was measured via a one-stage clotting assay (Sysmex CS-2500®) and compared with a standard reference curve.

#### IgG and albumin estimation

Serum samples were analyzed at the hematological laboratory, Small Animal Hospital, Faculty of Veterinary Medicine, Chiang Mai University. IgG and albumin concentrations were measured using a colorimetric assay (Sysmex BX-3010®, Kobe, Japan). The intra- and inter-assay coefficients of variation were 1.75% and 4.74%, respectively. All analyses were performed in duplicate under strict laboratory quality control, and all samples underwent only a single freeze–thaw cycle. The same technician processed all samples to minimize operator variability.

### Statistical analysis

Coagulation and protein concentrations were reported as mean ± standard deviation. A repeated-measures generalized linear model (GLM) was used to assess the effects of storage duration (0, 4, 8, and 12 months) on plasma protein stability. Model diagnostics included visual evaluation of Q-Q plots and residuals versus fitted values to confirm normality and homoscedasticity. Shapiro–Wilk tests were also performed. The GLM model was specified as: value ~ sex * month + (1 | elephantID). Post hoc pairwise comparisons were conducted using the multcompView package with Tukey-adjusted P values. Statistical significance was set at α = 0.05. All analyses were performed using R software (version 4.1.2; R Core Team, Vienna, Austria, 2023) [34].

## RESULTS

### Overall protein concentration patterns

The average concentrations of fibrinogen, factor VIII, IgG, and albumin in pre- and post-storage plasma samples stored at −20°C for 4, 8, and 12 months are summarized in Table 1. Statistical analyses showed that the month of collection was a significant predictor in all models, whereas sex did not significantly affect any coagulation or protein parameters (Table S1). Across storage durations, mean fibrinogen (F_3,54_ = 11.46, p < 0.001), factor VIII (F_3,54_ = 8.53, p < 0.001), IgG (F_3,54_ = 39.30, p < 0.001), and albumin (F_3,54_ = 26.87, p < 0.001) concentrations differed significantly.

**Table 1:** Mean±standard deviation plasma fibrinogen, factor VIII, serum IgG, and albumin before (month 0) and after frozen storage at 4, 8, and 12 months from captive Asian elephants (n = 20).

Parameter	Month			

	0	4	8	12
Fibrinogen (mg/dL)	255.16 ± 28.76^a^	264.37 ± 25.99^b^	248.63 ± 26.37^a^	248.69 ± 26.35^a^
Factor VIII (IU/dL)	900.89 ± 199.42^a^	833.24 ± 163.34^ab^	750.30 ± 170.72^b^	759.39 ± 162.24^b^
IgG (g/dL)	5.05 ± 0.47^a^	5.79 ± 0.65^b^	6.81 ± 1.09^c^	6.90 ± 1.14^c^
Albumin (g/dL)	3.31 ± 0.19^a^	3.29 ± 0.20^a^	3.58 ± 0.33^b^	4.02 ± 0.39^c^

^a,b,ab,c^ Different superscripts across rows indicate significant differences for each parameter (p < 0.05) using pairwise tests of independence (Tukey-adjusted).

### Fibrinogen stability during storage

Fibrinogen concentrations remained stable across storage periods. No significant differences were observed between fresh plasma and plasma stored for 8 months (248.63 ± 26.37 mg/dL, p = 0.165) or 12 months (248.69 ± 26.35 mg/dL, p = 0.172). These results indicate that fibrinogen remains stable when stored at −20°C for up to 12 months.

### Changes in factor VIII activity

Factor VIII activity declined markedly over time. Significant reductions were observed in samples stored for 8 months (750.30 ± 170.72 IU/dL, p < 0.001) and 12 months (759.39 ± 162.24 IU/dL, p < 0.001) compared with fresh plasma. Overall, factor VIII activity decreased by approximately 16% after 12 months of storage, though values remained within clinically acceptable limits.

### IgG concentration increases over time

Mean IgG concentrations increased substantially in frozen plasma samples across all storage durations. Significant differences were observed between fresh and stored plasma at 4 months (5.79 ± 0.65 g/dL, p = 0.003) and 8 months (6.81 ± 1.09 g/dL, p < 0.001), with a continued upward trend at 12 months (6.90 ± 1.14 g/dL, p < 0.001). By 12 months, IgG levels had increased by approximately 37%.

### Albumin concentration increases during storage

Albumin levels also increased significantly during storage. Compared with fresh plasma, albumin concentrations were higher at 8 months (3.58 ± 0.33 g/dL, p = 0.029) and at 12 months (4.02 ± 0.39 g/dL, p < 0.001). Overall, albumin levels rose by approximately 21% after 12 months of frozen storage.

### Graphical representation of trends

**Figure 1:**
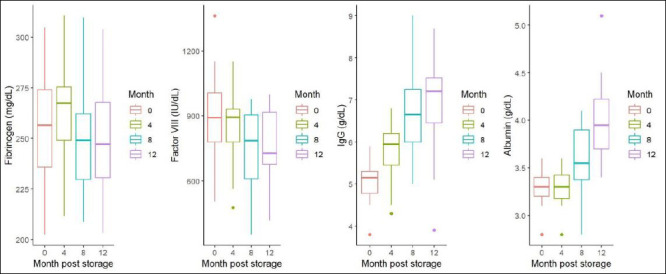
Average (a) fibrinogen, (b) factor VIII, (c) immunoglobulin G, and (d) albumin concentrations in pre- and post-storage plasma at –20°C for 4, 8, and 12 months from captive Asian elephants (n = 20).

The temporal trends in fibrinogen, factor VIII, IgG, and albumin concentrations across all storage intervals (0, 4, 8, and 12 months) are shown in Figure 1, illustrating the stability or variation of each parameter during −20°C storage.

## DISCUSSION

### Significance of the study and novel contribution

Unlike studies in canine and human medicine, this investigation represents the first controlled assessment of long-term protein stability in FFP derived from Asian elephants. These findings provide species-specific insights essential for developing emergency therapeutic strategies for EEHV-HD. Establishing evidence-based plasma storage guidelines is critical for creating the first plasma banking standards for captive elephants, enabling rapid intervention during hemorrhagic crises. Plasma transfusion plays a vital therapeutic role by supplying hemostatic proteins, mitigating hypovolemic shock, supporting immune function, and reducing the risks of red blood cell sensitization and volume overload. Although human FFP can be stored for up to 12 months at −20°C under American Association of Blood Banks guidelines, these standards may not directly apply to elephants because of species-specific differences in plasma protein stability.

### Stability of fibrinogen in frozen plasma

This study demonstrated a significant increase in fibrinogen concentrations after 4 months of frozen storage at −20°C, with non-significant decreases at 8 and 12 months compared with fresh plasma. These findings align with canine studies reporting stable fibrinogen levels after 30 days at −30°C [35] and with human studies showing no significant differences in fibrinogen when stored at −20°C or −70°C for 4 months [27]. Storage at ultra-low temperatures (−196°C) may provide even better preservation [27]. The Clauss assay used here is widely implemented in clinical settings [33], although discrepancies between Clauss and fibrinogen-derived methods have been documented [36, 37]. While freezing and storage can strongly influence PT and activated partial thromboplastin time (aPTT), fibrinogen appears less affected, with smaller changes reported at −70°C than at −20°C [27].

### Degradation of factor VIII during storage

Factor VIII concentrations decreased progressively during storage at −20°C, consistent with studies of canine plasma stored at −30°C for up to 12 months [26] and observations of declining factor VIII activity in canine plasma stored at −80°C [38]. As a labile protein, factor VIII is susceptible to deterioration during freezing, thawing, and long-term storage. The automated assay used may also contribute to measurement variability. Thawing at 37°C followed standard protocols [39], and prior studies show no detrimental effect on fibrinogen or factor VIII when thawed at 37–45°C [40] or across a wider temperature range (22°C–60°C) [41]. Despite the decline, factor VIII activity remained within clinically acceptable limits, supporting the potential clinical use of stored elephant FFP.

### Increases in IgG and albumin during storage

Storage at −20°C increased concentrations of IgG and albumin, likely due to cryoconcentration or subtle fluid loss during freezing. These elevated protein levels may prove beneficial in mitigating the hypoproteinemia characteristic of EEHV-HD. The successful adaptation of human-grade laboratory assays (Clauss assay, one-stage clotting assay, and colorimetric tests) to elephant plasma demonstrates methodological innovation and supports cross-species assay applicability.

### Comparison with previous research and methodological strengths

The selected storage durations (4, 8, and 12 months) were informed by prior canine FFP studies showing retained therapeutic activity after 3–12 months of storage [26]. Elephant plasma was aliquoted into small volumes (1–2 mL) to promote rapid freezing, in contrast to clinical plasma bags (200–250 mL), which freeze more slowly and may compromise factor stability. This study’s longitudinal design and use of repeated-measures GLM provide a rigorous statistical evaluation rarely achieved in wildlife transfusion research. Elephants may possess unique plasma protein stability characteristics, reflecting evolutionary adaptations in large mammals. Although comparative data are limited, the biological plausibility adds originality and relevance to the study.

### Study limitations and future directions

This study is limited by its modest sample size and assessment of a single storage temperature (−20°C). Future research should examine lyophilized [32, 42] and cryopreserved plasma at −80°C and −196°C, extend storage durations, and use larger aliquot volumes to better replicate clinical banking conditions. Additional clotting factors (e.g., II, VII, IX, X, and vWf) and coagulation parameters (PT, aPTT) should be evaluated to expand understanding of plasma stability. Clinical outcome studies involving EEHV-HD calves transfused with stored FFP are essential to validate therapeutic effectiveness.

### One health implications

Fibrinogen concentrations remained stable over 12 months of storage at −20°C, while factor VIII declined by 16% and IgG and albumin increased by 37% and 21%, respectively. Despite these changes, all parameters remained within clinically acceptable ranges for potential therapeutic use. Further investigations across varied storage conditions and longer durations are needed to refine species-specific guidelines. This study contributes to One Health efforts [43] by integrating wildlife medicine, transfusion science, and conservation biology to establish foundational protocols for elephant blood banking. The findings support the development of emergency FFP reserves for endangered megafauna, including rhinoceroses and tapirs, advancing conservation physiology and improving preparedness for critical care interventions.

## CONCLUSION

This study provides the first comprehensive evaluation of the long-term biochemical stability of FFP derived from Asian elephants and stored at −20°C, offering species-specific evidence essential for developing emergency transfusion protocols for EEHV-HD. The findings showed that fibrinogen concentrations remained stable for 8 and 12 months of frozen storage, while factor VIII activity declined by approximately 16% after 12 months yet remained within clinically acceptable ranges. In contrast, IgG and albumin concentrations increased significantly by 37% and 21%, respectively, after 12 months of storage, likely reflecting cryoconcentration effects. Collectively, these results indicate that elephant FFP retains adequate hemostatic and immunological function for up to 12 months at −20°C.

From a practical standpoint, the preservation of fibrinogen and acceptable factor VIII activity strongly supports the use of stored plasma to manage the acute hypoproteinemia and coagulopathy characteristic of EEHV-HD. The observed increases in IgG and albumin further suggest potential added benefits in supporting immune function and oncotic balance during emergency treatment. These findings establish scientifically grounded storage guidelines for elephant plasma banking, enabling wildlife hospitals and elephant conservation centers to maintain ready-to-use plasma reserves for life-threatening hemorrhagic events.

A major strength of this study is its controlled, longitudinal design, incorporating repeated-measures at 4, 8, and 12 months, combined with rigorous laboratory methodologies adapted from human medicine and validated for elephant plasma. The use of small-volume aliquots ensured rapid freezing and minimized degradation, providing an accurate assessment of plasma protein stability. This approach represents a methodological advancement rarely feasible in wildlife transfusion research.

In summary, storing frozen plasma at −20°C for up to 12 months preserves functional protein integrity suitable for clinical use in Asian elephants. These findings support the development of standardized blood banking systems for elephants, aid evidence-based clinical decisions during EEHV-HD outbreaks, and align with One Health approaches to enhance emergency care for endangered megafauna. Future studies should explore additional coagulation factors, different storage temperatures, and clinical outcomes to optimize species-specific transfusion protocols.

## DATA AVAILABILITY

The datasets generated and analyzed in this study are available upon reasonable request from the corresponding author.

## AUTHORS’ CONTRIBUTIONS

CT, PB, JLB, and PV: Designed the research concept and methodology. AP, KA, CS, and PV: Conducted laboratory work. CT, KA, and SK: Collected samples. KA, SK, PB, and WK: Analyzed the data. CT, AP, and KA: Wrote the first draft of the manuscript. CT, PB, WK, JLB, and PV: Reviewed and edited the manuscript. All authors have read and approved the final version of the manuscript.
